# Orchestrating multiple signal inputs: conducting secondary differentiation in the obligate intracellular bacterium *Chlamydia trachomatis*

**DOI:** 10.1128/mbio.01031-26

**Published:** 2026-07-20

**Authors:** Vandana Singh, Derek J. Fisher, Scot P. Ouellette

**Affiliations:** 1 Department of Pathology, Microbiology, and Immunology, College of Medicine, University of Nebraska Medical Center 198515, Omaha, Nebraska, USA; 2 School of Biological Sciences, Southern Illinois University 2254, Carbondale, Illinois, USA; The Ohio State University, Columbus, Ohio, USA

**Keywords:** *Chlamydia*, development, differentiation, signaling, redox, ATP, protease

## Abstract

*Chlamydia trachomatis* is a gram-negative obligate intracellular pathogenic bacterium that is a leading cause of sexually transmitted and ocular infections. Pathogenic *Chlamydia* species have a distinct, essential, and tightly controlled biphasic developmental cycle. The non-replicative elementary body (EB) infects a cell and undergoes primary differentiation to the non-infectious reticulate body (RB), which undergoes multiple rounds of division. The shift from the replicative RB form to its infectious EB form, known as secondary differentiation, is a crucial step in this cycle and occurs asynchronously during later stages of development. This process is essential for the pathogen’s ability to spread between host cells and to be transmitted between hosts. Although its importance is clear, the molecular mechanisms behind this key transition are not well understood. In this review, we outline the current knowledge of how *C. trachomatis* transitions from the RB to the EB. We focus on aspects of secondary differentiation where significant mechanistic insights have been uncovered. Emerging evidence suggests that secondary differentiation is regulated by a network of noncanonical pathways, including controlled proteolysis, redox-dependent signaling, cyclic di-AMP, ATP sensing, and sigma factor-mediated transcription. This review examines the cellular and molecular cues that drive secondary differentiation in *C. trachomatis* and discusses their implications for pathogenesis and potential treatments. The goal is to provide a current and comprehensive overview of secondary differentiation and its role in understanding chlamydial disease.

## INTRODUCTION

*Chlamydia trachomatis* is an obligate intracellular pathogen responsible for the most common bacterial sexually transmitted infection, chlamydia, and the ocular infection known as trachoma. It has a distinctive biphasic developmental cycle consisting of two morphological forms: the elementary body (EB) and the reticulate body (RB) (1). For *C. trachomatis* serovar L2, a genetically tractable strain commonly used in the lab, the developmental cycle takes ~48-54 h. The cycle begins when an EB infects a host cell. Inside a specialized, membrane-bound parasitic organelle called the inclusion, the EB quickly differentiates into the RB-a process known as primary differentiation. After multiple rounds of replication, RBs asynchronously differentiate into EBs, referred to as secondary differentiation, via an intermediate body (IB). Secondary differentiation is crucial for chlamydial spread within and between hosts and results in disease development. To fully understand how *Chlamydia* sustains infection, it is vital to understand the mechanisms behind secondary differentiation. Such understanding could also lead to new therapeutic strategies to disrupt its complex developmental cycle, as current treatments rely on broad-spectrum antibiotics that disrupt normal flora, resulting in susceptibility to other infections.

EBs and RBs differ significantly: EBs are infectious, non-replicative, and smaller (~0.3 µm), while RBs are non-infectious, capable of replication, and larger (~1 µm). Early work from Tamura and Manire demonstrated differences in the overall chemical composition of EB and RB membranes (2, 3). This was followed by electron microscopy studies by Tamura and Matsumoto that showed structural differences between developmental forms (4). Besides the morphological and functional differences of EBs and RBs, multiple studies have attempted to characterize gene expression and other differences between these developmental forms. Seminal work from the Hatch lab, for example, identified stage-specific transcripts expressed early after infection (5) and later during the developmental cycle (6). This was followed by work from the Hackstadt lab, who showed three clear patterns of transcription (early, mid, and late) during the developmental cycle using RT-PCR of a handful of target genes (7). Early-stage genes are presumed important for primary differentiation and establishment of the inclusion; mid-stage genes are presumed important for RB growth and division and further modifying host cell function; late-stage genes are presumed important for secondary differentiation and pre-loading the EB with effectors necessary for infection of a host cell. These targeted studies were later extended to the whole genome by Belland et al. using microarray analyses (8). On the protein side, work from the Hatch lab (9–11), Schachter lab (12), and Hackstadt lab (13–16) identified factors modified or expressed only in the EB. Key among these were the major outer membrane protein (MOMP), the cysteine-rich outer membrane complex proteins OmcA and OmcB, and several heat-stable structural proteins that contribute to the EB’s disulfide-cross-linked envelope, thereby contributing to the rigidity and environmental stability of EBs. Two histone-like proteins, Hc1 and Hc2, were identified in EBs. These findings clarified the characteristic features of EBs, including their compacted chromatin, the molecular basis of infectivity, and EB antigenicity.

**Fig 1:**
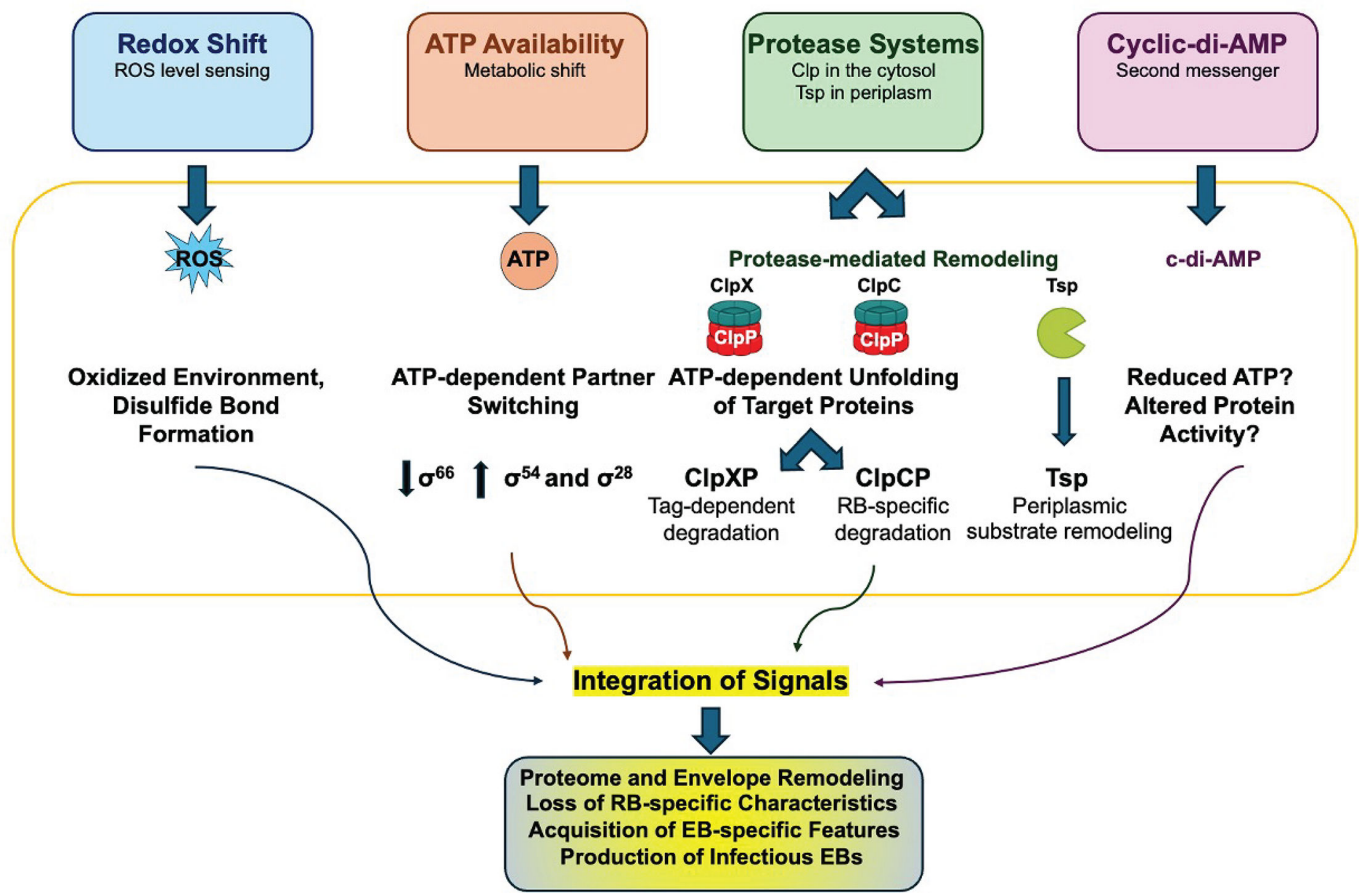
Integrated signals, including redox state, cyclic di-AMP, ATP availability, and protease activity, drive secondary differentiation. Oxidizing conditions promote disulfide bond formation, while cyclic di-AMP acts as a second messenger. ATP links nutrient and energy status to developmental progression, regulating ATP-dependent partner switching and σ-factor activity, coordinating a transcriptional cascade involving σ⁶⁶ (RB maintenance), σ⁵⁴ (developmental activation), and σ²⁸ (EB gene expression). In parallel, AAA+ unfoldases (ClpC, ClpX) and proteases (Tsp, ClpXP, and ClpCP) mediate protein remodeling and degradation. Interplay of these processes drives proteome and envelope remodeling, resulting in loss of RB characteristics and acquisition of EB features, including a disulfide-rich outer membrane, culminating in infectious EB formation.

Although much work has been conducted to identify differences between EBs and RBs, it remains largely unknown how differentiation between these forms is achieved. Recent work from our labs and other groups has sought to dissect this essential step in chlamydial development. While most published work has focused on transcriptional regulation of secondary differentiation, this review explores post-translational mechanisms that control secondary differentiation with a particular emphasis on regulated proteolysis, redox status, cyclic di-AMP signaling, and cellular energy levels, which determine the timing and accuracy of this process. We discuss recent findings to explain how these interconnected pathways coordinate environmental, metabolic, and stress signals to promote asynchronous RB to EB differentiation (Fig. 1), underscoring their vital roles in the chlamydial developmental cycle and pathogenicity.

## POST-TRANSLATIONAL MECHANISMS ORCHESTRATING SECONDARY DIFFERENTIATION

In *Chlamydia*, the process of secondary differentiation is complex and can be influenced by several signals. We discuss the effects of these signals on secondary differentiation as follows.

### Cytoplasmic and periplasmic proteases

In *C. trachomatis*, multiple proteomic analyses have demonstrated that EBs and RBs possess distinct protein repertoires while showing differences in overall abundance of shared proteins (17–20). These studies indicate that protein abundance is linked to the specific needs of each developmental form. For example, RBs (the form involved in growth and cell division) contain abundant proteins related to translation, chaperones, cell division, redox balance, and nucleic acid metabolism. In contrast, EBs (the extracellular form involved in infectivity) are characterized by the abundance of proteins associated with the cell envelope to support extracellular survival and infection, and by energy metabolism to maintain EB viability outside the cell and fuel early post-infection events. Importantly, the transition between RBs and EBs is not driven by conventional cell division processes that selectively redistribute existing intracellular proteins to two different types of cells as occurs, for example, during the *Caulobacter* developmental cycle (21). Instead, differentiation from one form to another occurs post-division and is likely dependent on extensive protein turnover involving the degradation of stage-specific (i.e., RB) proteins to support functional and morphological remodeling of the bacterium in concert with new gene expression (i.e., late genes or EB-specific proteins). Protein turnover between RBs and EBs is performed by cytoplasmic proteases, such as the caseinolytic protease (Clp), and periplasmic proteases, such as the tail-specific protease (Tsp). Our group has provided evidence that this protein turnover is critical for secondary differentiation, with the cytosolic proteases ClpXP and ClpCP and the periplasmic protease Tsp serving as key mediators of secondary differentiation in *Chlamydia* (22–26).

The Clp system is a complex of proteins that work together to recognize and degrade misfolded or damaged proteins as well as specific substrates via degron-tagging or chaperone-mediated delivery. Clp proteases serve important functions in protein homeostasis, stress, and pathogenesis (27). In spite of its reduced genome size, *C. trachomatis* encodes multiple Clp protease components, including two AAA+ unfoldases (ClpX and ClpC), which recognize and unfold substrates, and two ClpP peptidase subunits (ClpP1 and ClpP2), which degrade substrates (28). In *C. trachomatis,* ClpP2 is part of an operon with ClpX, whereas both ClpP1 and ClpC are found in chromosomally distinct regions. In the ClpXP or ClpCP protease system, ClpX or ClpC forms a hexameric ring that recognizes and unfolds target proteins using ATP to feed them into ClpP, a proteolytically active, barrel-shaped tetradecameric complex that facilitates their degradation (29, 30). Biochemical analyses of chlamydial ClpX and ClpC confirmed their ATPase activity and assembly into a homohexameric ring *in vitro*, supporting their roles in unfolding substrates (23, 26). ClpP1 and ClpP2 each form homoheptamers, but it remains unclear how ClpP interacts with the unfoldases in *Chlamydia*. Active complexes may require both a ClpP1 heptamer and a ClpP2 heptamer (26, 31), but crystal structures of ClpP2 indicate a homotetradecameric complex (32). Further work is required to determine the *in vivo* composition of the Clp protease complexes. Additional research has shown distinct effects of disrupting ClpP1 and ClpP2 on chlamydial growth. Overexpression of a catalytic mutant of ClpP1 (S92A) severely disrupted chlamydial growth at 24 h post-infection (hpi), whereas a negative impact of overexpressing a catalytic mutant of ClpP2 (S98A) was not apparent until 48 hpi (23, 33).

Because the unfoldases are the gatekeepers of the Clp protease systems, we have investigated their function in chlamydial growth and development. In regard to ClpX, we have focused on two key residues: E187 and R230. The E187 residue is within the Walker B ATPase domain, and mutating this residue (E187A) abolishes ATPase activity and the ability of ClpX to unfold substrates (23). The R230 residue is part of the RKH motif critical for recognizing SsrA-tagged proteins and, when mutated (R230A), blocks recognition of such tagged proteins (22). The SsrA tag, encoded by *tmRNA*, is a degron that is added to partially translated peptides generated by the *trans*-translation pathway, which rescues stalled ribosomes on mRNA transcripts lacking a stop codon. Consequently, SsrA-tagged proteins are directed to ClpXP (or other proteases like Lon) for degradation.

When assessing the effects of overexpressing the E187A, R230A, or double E187A/R230A ClpX isoforms in *C. trachomatis*, we observed notable impacts on chlamydial growth and development. Although overexpressing the ClpX E187A isoform had no statistically significant effect on replication or expression of EB-specific markers, it did severely impact infectivity and morphology of EBs (23). Similar effects were noted with overexpressing the E187A/R230A ClpX isoform (22), establishing that the ATPase activity is epistatic to the RKH domain. Interestingly, overexpressing the R230A mutant caused a defect in secondary differentiation but did not significantly impact bacterial replication. While superficially similar to the E187A phenotype, RB to EB formation was blocked even though transcripts of late-cycle genes remained unaffected. This finding challenges the idea that transcriptional activation of late-cycle genes alone is enough to induce secondary differentiation. To further dissect the function of *trans*-translation in chlamydial growth and development, a CRISPRi knockdown strain targeting *tmRNA* was generated and assessed. Knockdown of *tmRNA* disrupted chlamydial developmental cycle progression, indicating an essential function of *trans*-translation consistent with effects of antibiotics targeting this process (22). Complementing *tmRNA* knockdown with a wild-type copy of *tmRNA* rescued the negative impact of knocking down *tmRNA*. However, expressing a *tmRNA* allele encoding a 6xHis tag in place of the SsrA degron sequence, meaning ribosomes could be rescued but the non-stop translated product not degraded, phenocopied the effects of overexpressing the R230A ClpX isoform (i.e., replication was restored but not secondary differentiation). These data indicate that the inability to degrade SsrA-tagged substrates blocks developmental progression from RB to EB. Based on these findings, we proposed a model in which the buildup of SsrA-tagged substrates shifts the substrate preference of ClpX from a factor(s) necessary for promoting secondary differentiation to the SsrA-tagged pool of substrates (22). This, in turn, leads to the availability of the factor(s) to trigger secondary differentiation. Overall, these data support an essential function of ClpX in mediating secondary differentiation (Fig. 1).

Unlike ClpX, which is highly conserved across all eubacteria, ClpC is generally limited to gram-positive bacteria and mycobacteria. Like other ClpC orthologs, the chlamydial ClpC retains the essential residues required for its ATPase and unfoldase activities, including both nucleotide-binding domains (NBD1 and NBD2) of the Walker B motif, along with residues required to recognize and degrade phosphoarginine-marked proteins. Similar to ClpX, chlamydial ClpC forms a functional protease by associating with the heteromeric ClpP1P2 complex *in vitro*. ClpC specifically interacts with this complex via ClpP2 to degrade select substrates and also appears to possess ATPase-dependent chaperone activity (26). More recently, we characterized the effects of overexpression of wild-type or ATPase-null ClpC isoforms on chlamydial growth and development. Overexpression of ATPase-null ClpC mutants resulted in reduced EB production, but the inclusion morphology remained normal. In contrast, overexpression of the wild-type ClpC isoform resulted in much smaller inclusions and a significant reduction in EB production at 24 hpi. This is markedly different from the effects of overexpressing wild-type ClpX or ClpP1/2, where no negative effects on chlamydial growth were measured. Further investigation of the effects of wild-type ClpC overexpression revealed interesting phenotypes, such as premature glycogen accumulation (a marker of late-stage development), earlier expression of late-cycle and other EB-associated transcripts, and increased EB yields at earlier timepoints in the developmental cycle. Collectively, these phenotypes indicate an earlier onset of infectivity and accelerated secondary differentiation when overexpressing wild-type ClpC (25).

**Fig 2:**
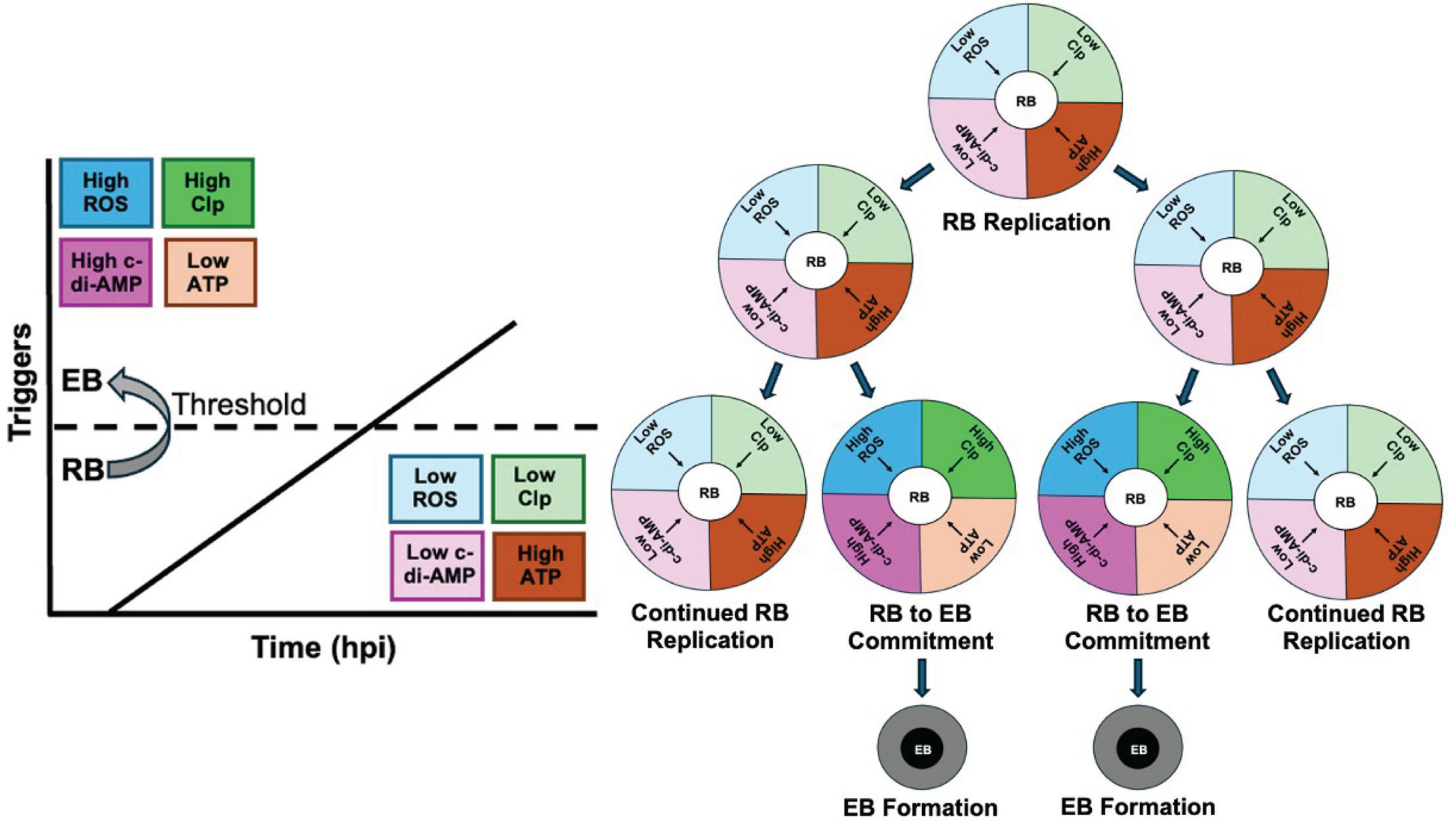
Threshold model for signal-dependent regulation of RB maintenance and secondary differentiation in *Chlamydia trachomatis*. RB replication versus secondary differentiation is governed by at least four signals: redox state, cyclic di-AMP, ATP availability, and protease activity. In the RB state, a reduced intracellular redox environment, low cyclic di-AMP, low Clp activity, and relatively high ATP levels favor continued growth and division of RBs. As infection progresses, pro-differentiation triggers accumulate, including increased ROS, elevated c-di-AMP, increased Clp activity, and reduced ATP availability. These inputs are integrated over time and must cross critical thresholds before the RB commits to EB (secondary differentiation), activating downstream remodeling pathways and conversion into infectious EBs. The graph illustrates the proposed increase in integrated differentiation triggers over time, with RB replication favored below the threshold and EB formation favored upon crossing the threshold. Color intensity indicates relative signal strength, and the dashed line represents the proposed threshold separating RB maintenance from EB differentiation.

This study suggests that ClpC is essential for secondary differentiation since altering its levels or activity disrupts developmental cycle progression. The data support a model in which increased ClpCP proteolytic activity beyond a critical threshold triggers the transition from RBs to EBs (Fig. 2). Conversely, impairing the function or level of ClpC delays this transition. These findings establish ClpC as a key molecular regulator of the RB- to EB transition, with its ATPase activity required to drive the proteolytic remodeling needed for differentiation. This probably happens through the degradation or chaperone-assisted restructuring of substrates that maintain the RB state, thereby allowing the activation of EB-specific programs. ClpC’s ability to regulate the timing of developmental progression suggests it functions as a regulatory checkpoint, coordinating proteostasis with developmental signaling in *Chlamydia*. Excitingly, small molecule compounds that alter the activity of ClpP or ClpX inhibit chlamydial growth, highlighting how proteins associated with developmental regulation can be therapeutic targets (34).

*Chlamydia*, as a gram-negative bacterium, has four compartments: cytoplasm, inner membrane, periplasm, and outer membrane. In addition to cytoplasmic proteome turnover by Clp proteases, periplasmic protein turnover is also critical for secondary differentiation in *Chlamydia. C. trachomatis* encodes two main periplasmic proteases: HtrA and Tsp. HtrA, a mid-cycle gene, has been characterized as an essential periplasmic protease during the mid-replicative phase of the chlamydial developmental cycle and is crucial for bacterial stress response under conditions such as heat shock, penicillin, or IFNγ-induced persistence (35, 36). Tsp is a homolog of bacterial tail-specific protease (aka Prc). In gram-negative bacteria, Tsp/Prc participates in periplasmic homeostasis by degrading misfolded proteins, peptidoglycan-associated proteins, and SsrA-tagged proteins. Unlike most bacteria, where *prc/tsp* expression is constitutive, chlamydial *tsp* is a late-cycle gene, and its regulation by σ^28^, along with another key late-cycle gene, *hctB,* indicates its role in secondary differentiation (37).

Our group’s functional analysis further confirmed that Tsp plays a crucial role as a mediator in chlamydial secondary differentiation (24). Overexpression of wild-type Tsp (but not a catalytically inactive S455A mutant) was highly harmful to the progression of the chlamydial developmental cycle by blocking RB replication and hindering the subsequent production of EBs. Conversely, CRISPRi-mediated reduction of Tsp activity had little effect on RB replication but did impact infectious progeny production. The number of infectious progeny was severely decreased, although there was no effect on the expression of late-cycle genes such as *hctB* or *omcB*. Transmission electron microscopy (TEM) showed a void space either between the inner and outer membranes or between the nucleoid and the inner membrane, leading to the production of morphologically abnormal and non-infectious EBs (24). The different effects observed in Tsp overexpression and knockdown suggest that Tsp may be involved in degrading or facilitating the degradation of proteins involved in chlamydial peptidoglycan synthesis and in converting RBs to EBs (Fig. 2). Additionally, Tsp may be necessary during secondary differentiation to specifically eliminate SsrA-tagged proteins in the periplasm, preventing the buildup of potentially toxic peptide fragments in the EB. Another study found that a *Chlamydia muridarum tsp* nonsense mutant derived via chemical mutagenesis was deficient in infection at 37°C and completely blocked at 40°C (38). The authors suggested that Tsp is involved in degrading chlamydial outer membrane complex proteins, such as OmcA and OmcB, during the late stage of the chlamydial developmental cycle (38). Overall, the data show that the activity of Tsp is essential for the formation of mature, functional EBs; without it, the EBs cannot revert to RBs upon entering and infecting a new host cell.

### Redox regulation

Another major difference between EBs and RBs is their relative redox status (39). A study from Wang et al. used a redox-sensitive GFP reporter expressed in different compartments of the host cell or *Chlamydia* itself to explore the redox state of the host cell and bacteria during the developmental cycle. Their data revealed that, during the transition from RB to EB (secondary differentiation), the redox status shifts from reduced to oxidized (Fig. 2). Notably, the redox status of host cells remains unchanged, indicating that redox modulation is intrinsic to *Chlamydia* (39). These findings are consistent with earlier studies in the field examining the redox status of different proteins/systems in *Chlamydia* as well as the known characteristics of the pathogen. For example, the oxidized environment in EBs is necessary for the highly disulfide-crosslinked chlamydial outer membrane proteins, including the major outer membrane protein (MOMP), OmcA, and OmcB, and components of the type III secretion system (T3SS), such as CdsF, CdsD, and CdsC (40–42). Relatedly, studies have shown that cysteine depletion affects chlamydial growth and the differentiation of RBs into EBs, likely due to impaired disulfide bond formation (10, 43). This crosslinking is crucial not only for maintaining the structural rigidity and stability of EBs but also for their function during infection of a host cell. Treatment of EBs with the reducing agent dithiothreitol (DTT) caused changes in the cell wall typical of RBs and was associated with lower infectivity, reduced osmotic stability, and altered staining patterns (44). Consequently, changes in bacterial redox states are essential for chlamydial development (Fig. 1).

In gram-negative bacteria, disulfide bond formation depends on two key proteins, DsbA and DsbB. DsbA, a member of the thioredoxin superfamily, introduces disulfide bonds into substrate proteins and becomes reduced in the process. DsbB then reoxidizes DsbA through quinone-dependent electron transfer. This reoxidation involves conserved periplasmic cysteine residues in DsbB that form transient mixed disulfide intermediates with DsbA, completing the oxidative folding cycle (45–48). Despite its reduced genome, *Chlamydia* retains a canonical Dsb oxidative folding pathway similar to that of *Escherichia coli,* underscoring the critical importance of disulfide bond formation in chlamydial biology. Chlamydial DsbA functions as a thiol-disulfide oxidoreductase. It contains a conserved Cys-X-X-Cys catalytic motif and adopts the classical thioredoxin-like DsbA fold. However, its redox potential is less oxidizing than that of *E. coli* DsbA, indicating comparatively weaker oxidative power. Structural studies further revealed unique features, including additional cysteine residues and altered surface charge, which likely influence its redox properties (49). In 2019, Christensen and colleagues characterized the chlamydial DsbB, encoded in an operon with *dsbA*, and demonstrated the role of the redox pair DsbA and DsbB in disulfide bond formation in *Chlamydia* (50). The *dsbAB* operon is transcribed as a late-cycle gene, consistent with a function in secondary differentiation, and DsbA is expressed during the RB to EB transition, coinciding temporally with the progressive oxidation of the chlamydial outer membrane complex. The timing of expression and functional redox pairing between DsbA and DsbB indicates their direct role in secondary differentiation and increasing oxidation in the chlamydial cell wall (50).

What is the cause of increasing oxidation within the RB itself? We hypothesize this shift in the redox state is linked to the metabolic activity of RBs and the buildup of reactive oxygen species (ROS) as byproducts of metabolism, such as oxidative phosphorylation. To test this, we explored the function of the primary antioxidant, alkyl hydroperoxidase reductase subunit C (AhpC), as a scavenger of reactive oxygen species in chlamydial growth and development (51). Genetically modifying the expression levels of AhpC led to different sensitivities to oxidizing agents and the expected changes in redox state. With CRISPRi-mediated reduced levels of AhpC, intracellular ROS levels increased, creating a more oxidized environment, with bacteria more sensitive to oxidizing agents. In this more oxidized state, transcripts of EB-associated genes and higher EB production appeared *earlier* in the developmental cycle, indicating that an oxidized environment promotes secondary differentiation (Fig. 2) (51). Conversely, overexpression of AhpC produced a more reducing environment with bacteria displaying enhanced resistance to oxidizing agents, and this led to an extended replicative phase of RBs and delayed transition from RB to EB (51). These results support a redox threshold model, in which, during RB growth and division, AhpC maintains the oxidation level below a threshold by scavenging ROS, allowing RBs to continue dividing and replicating. When ROS production exceeds AhpC’s capacity to scavenge ROS, the oxidation level surpasses the threshold, triggering RB to EB differentiation (51). Since secondary differentiation is an asynchronous process, and RBs divide via a polarized budding mechanism (52, 53), we speculate that the accumulation of ROS causes uneven distribution of oxidized proteins during division in RBs, with one daughter cell “inheriting” more oxidized proteins than the other. Such a daughter cell may breach the oxidative threshold earlier than the other and initiate secondary differentiation, as indicated by earlier detection of EBs and associated late-cycle genes (Fig. 2). This study provides direct evidence for the role of redox homeostasis in the chlamydial developmental cycle (51). Ongoing studies are designed to identify redox-regulated proteins in *Chlamydia* to better understand how increased oxidation impacts protein function to trigger secondary differentiation.

### Cyclic di-AMP

Cyclic di-AMP (c-di-AMP) is a well-recognized second messenger in gram-positive bacteria and has been linked to osmotic homeostasis, DNA repair, and sporulation (54, 55). Its levels are regulated through three mechanisms: synthesis by diadenylate cyclase (DacA), degradation by phosphodiesterase (PDE), and secretion via transporters (56). Though *Chlamydia* is a gram-negative bacterium, it encodes diadenylate cyclase (*dacA*) in an operon with its regulator (*ybbR*), but it lacks any annotated homolog of PDE or putative c-di-AMP transporters (28). In 2013, a study from Barker et al. sought to understand how chlamydial infection activates IFNβ production. The authors determined this occurred via the innate immune response protein STING, which detects chlamydial c-di-AMP (57, 58). They further showed that c-di-AMP levels increase over the developmental cycle and that EBs have higher levels than RBs (Fig. 2). DacA diadenylate cyclase activity was confirmed *in vitro*, and a mutant isoform (D164N) displayed significantly reduced c-di-AMP production. However, the function of c-di-AMP in chlamydial physiology was not explored.

Given that an increase in c-di-AMP levels coincides with RBs differentiating into EBs, we hypothesized that c-di-AMP is a signal for secondary differentiation. To test this, c-di-AMP synthase genes (*dacA* and *ybbR*) were knocked down or overexpressed, and phenotypes were examined to determine how different c-di-AMP levels affect secondary differentiation of *Chlamydia* (59). Using these genetic approaches, the ability to modulate c-di-AMP production in *Chlamydia* was established. A lower level of c-di-AMP resulted in fewer EBs being produced without affecting genome copy numbers, which represent both RBs and EBs. Lower EBs with unchanged genome copy numbers suggest that reduced c-di-AMP levels maintain RBs but inhibit or delay conversion to EBs, thereby postponing secondary differentiation. Overexpression of the *dacA* operon (*dacA* and *ybbR*) produced significantly higher levels of c-di-AMP, leading to earlier production of EBs along with increased expression of canonical and EB-associated late-cycle genes. This study identified c-di-AMP as an internal signaling molecule in *Chlamydia* and indicated that a threshold level of c-di-AMP is needed to trigger RBs to mature into EBs (Fig. 2).

This study also established that YbbR is essential for DacA function, and the stoichiometry between DacA and YbbR is critical for c-di-AMP signaling activity. The overexpression of *dacA* alone increased c-di-AMP levels approximately fourfold, but this increase was below the levels observed at 24 h post-infection when EBs are being produced and was insufficient to trigger earlier EB formation. The overexpression of *dacA* disrupted chlamydial growth and development, evidenced by smaller inclusion size, reduced EB production, and decreased genome copy numbers. Overexpression of the DacA active-site mutant isoform, D164N, alone severely impaired c-di-AMP production, confirming the requirement of an intact catalytic center for nucleotide synthesis. Strikingly, this strain still decreased inclusion size, EB production, and genome copy number. This suggests that dysregulated DacA expression disrupts developmental progression independently of absolute c-di-AMP concentration, potentially through dominant-negative or membrane-associated effects (58, 59). Excess membrane insertion of DacA in the absence of sufficient YbbR may disrupt RB membrane integrity or protein interactions. Consistent with this model, overexpression of ΔTM (transmembrane) isoforms of either wild-type or D164N DacA had no detrimental effects on growth, and ΔTMDacA (D164N) did not alter c-di-AMP levels. These findings indicate that membrane localization and YbbR interaction are central to DacA function. The dominant-negative phenotype observed with membrane-bound DacA (D164N) likely results from interference with wild-type DacA activity, possibly through hetero-oligomer formation or sequestration of YbbR. In contrast, transcript knockdown of *dacA-ybbR* reduced but did not eliminate protein levels, leading to residual c-di-AMP production and milder phenotypes compared to mutant overexpression strains (59).

Several potential mechanisms may regulate YbbR activity and thereby modulate c-di-AMP levels. As a monotopic membrane protein, YbbR could be subject to proteolytic degradation by inner membrane-associated proteases such as FtsH or by periplasmic proteases including HtrA and Tsp. Proteolysis of YbbR would effectively shut down DacA activation and reduce c-di-AMP synthesis. Alternatively, YbbR may facilitate interactions between membrane-bound DacA and additional membrane partners; overexpression of DacA alone could destabilize these complexes and impair enzymatic function. An additional layer of regulation may involve cellular energy balance. Because DacA synthesizes c-di-AMP from ATP, elevated DacA activity could reduce intracellular ATP pools (Fig. 1). Decreased ATP availability may lower σ^66^-dependent transcriptional activity, potentially promote earlier initiation of EB-associated gene expression, and accelerate secondary differentiation (see below). This links nucleotide signaling directly to metabolic state and transcriptional control. These possibilities underscore a multilayered regulatory system involving protein–protein interactions, membrane context, and proteolytic control. As with redox regulation, the identification of c-di-AMP binding proteins is essential to understand how c-di-AMP is impacting secondary differentiation in *Chlamydia*. Alternatively, c-di-AMP could bind riboswitches that may trigger changes in gene expression. Clearly, further investigation is required to better understand the function of c-di-AMP in *Chlamydia*.

### Sigma factors and ATP

Metabolic signals, especially ATP levels and sigma-factor-mediated transcriptional regulation, are two key features of the chlamydial developmental cycle; together, they form a metabolic-transcriptional axis that guides the timing and progression of secondary differentiation. Three sigma factors, σ^66^, σ^54^, and σ^28^, regulate the precise timing of gene expression in *Chlamydia* during its developmental cycle (7, 8, 60). While σ^66^ functions as the main housekeeping sigma factor, the other two, σ^54^ and σ^28^, are active during the later stages of development (37, 60, 61). σ^54^ initiates a transcriptional cascade by activating genes involved in membrane construction, type-III secretion systems critical for EBs, and other late-cycle genes (37, 62). The σ^54^ is epistatic to σ^28^ and is a necessary precursor for the endogenous activity of σ^28^. σ^28^ has a very restricted regulon comprising *hctB* and *tsp*-two late-cycle genes essential for EB maturation (37). HctB and Tsp drive significant changes in chromosomal and periplasmic structure during secondary differentiation and require precise regulation of transcriptional activation to avoid detrimental defects (16, 24). Altering the abundance of σ^54^, σ^28^, or σ^66^ causes severe defects in EB formation, underscoring their vital roles in the chlamydial developmental cycle (37). Knockdown of either alternative sigma factor or their combined depletion greatly reduces elementary body production. The knockdown strains appear mostly normal, yet exhibit a marked decrease in EBs, indicating that these sigma factors are not essential for early or mid-cycle RB growth but become critical during late-cycle development. Conversely, overexpression of σ^54^, σ^28^, or both leads to enlarged RBs and a sharp decline in EBs, further confirming that, while these factors are not needed for RB production and maintenance, their premature expression disrupts developmental cycle progression. These data support that the ratio of the three sigma factors during the developmental cycle is important for chlamydial growth.

To control sigma factor activity, many bacteria utilize a protein phosphorylation-based signal transduction system called the partner switching mechanism (PSM), involving cycles of phosphorylation and dephosphorylation (63–65). PSMs facilitate rapid transcriptional reprogramming, aiding adaptation and survival. In *Chlamydia*, the lone PSM includes two sensor phosphatases (RsbU and an uncharacterized RsbU-like protein CTL0852), an anti-sigma factor with kinase activity (RsbW), and anti-anti-sigma factors (RsbV1/RsbV2), which serve as substrates for both RsbW and RsbU (60, 66, 67). The Rsb-PSM in *Chlamydia* functions as a metabolic sensor, linking the organism’s metabolic status to its developmental cycle. Relevant to the chlamydial PSM, while *Chlamydia* can carry out oxidative- and substrate-level phosphorylation, these bacteria are auxotrophic for nucleotides that are parasitized from the host cell via two nucleotide transporters, Npt1 (ATP/ADP/NAD translocase) and Npt2 (NTP uniporter (68, 69). In addition, *C. trachomatis* has a partial TCA cycle (28, 70) and can import TCA intermediates from the host cell, and they import host cell-derived glucose-6-P for glycolysis. The interaction between RsbW and RsbV1/RsbV2 depends on ATP, while RsbU can sense host-derived TCA cycle intermediates such as α-ketoglutarate, malate, and oxaloacetate, and RsbU phosphatase activity is inhibited by the glycolysis intermediate phosphoenolpyruvate (65, 71, 72). Micronutrients may also play a role in PSM regulation, as RsbU phosphatase activity is enhanced in the presence of Mn^2+^ compared to Mg^2+^, although changes in metalation levels between the RB and EB have largely not been explored (72–74). Collectively, these interactions allow the PSM to be responsive to host cell and bacterial metabolites.

ATP is essential throughout the chlamydial developmental cycle: it is required in the early stages by EBs, for biosynthetic processes in RBs, and as a regulatory signal in the transcriptional network (75, 76). Although it is not yet clear whether TCA ligands activate or inhibit RsbU’s phosphatase activity, recent reports suggest that during the mid-stage of the developmental cycle, when ATP and TCA metabolites are abundant, RsbW phosphorylates RsbV1/RsbV2, which are then dephosphorylated by ligand-bound RsbU. This cycle releases σ^66^ from RsbW, ensuring that σ^66^ activity reflects *Chlamydia*’s metabolic state and supports replication when nutrients and energy are plentiful (Fig. 2). As the cycle advances, nutrient depletion, including ATP and α-ketoglutarate, occurs due to the increasing number of RBs, leading to less efficient RsbU dephosphorylation of P-RsbV1/P-V2 (phosphorylated forms cannot bind RsbW) and reduced interactions between RsbW and RsbV1/V2 (requires ATP). This results in increased sequestration of σ^66^ by RsbW (71, 77). Consequently, this regulatory switch allows alternative sigma factors, σ^28^ and σ^54^, to access the RNA polymerase core, promoting their binding and activating the expression of late-cycle genes (Fig. 1). Supporting this model, a *C. trachomatis* RsbU null mutant (excessive RsbW sequestration of σ^66^) is severely impaired for growth and EB production and has decreased transcription of σ^66^-dependent genes (71), while a strain overexpressing a non-phosphorylatable RsbV2 mutant (RsbW trap, excessive free σ^66^) exhibits normal levels of bacteria (gDNA) but a 3-log reduction in EB production and reduced levels of late-stage proteins Tarp and HctB (77). Through this dynamic interaction, the chlamydial Rsb-PSM system acts as a regulatory node linking metabolic cues to transcriptional regulation, thus coordinating developmental progression, including the critical transition from RBs to EBs (77). Collectively, these studies demonstrate that ATP and metabolite availability, sigma factors (σ^28^ and σ^54^), and the PSM are key regulators of secondary differentiation.

## SIGNAL INTEGRATION DURING CHLAMYDIAL SECONDARY DIFFERENTIATION

Since multiple systems can impact chlamydial secondary differentiation timing, this suggests that *Chlamydia* carefully integrates different signal inputs to ensure its intrabacterial conditions are optimal for transitioning from the non-infectious RB to the infectious EB. This is an essential step in its growth and pathogenesis. The developmental cycle effectively proceeds like a clock, and *Chlamydia* has evolved various systems to allow for stochasticity such that individual organisms can differentiate asynchronously when conditions are appropriate (Fig. 2). This likely ensures a population of infection-competent EBs to spread infection as soon as possible in the event that their host cell is compromised while allowing for a larger population of EBs to accumulate to maximize spread. If too many RBs transition too soon, then there will be fewer EBs to propagate the infection, which exposes *Chlamydia* to elimination by the immune response. If EB formation happens too late, then host cell lysis will result in non-infectious RBs being released. This bet-hedging strategy is distinct from mechanisms used by other bacteria growing within a community, specifically those using quorum sensing. *Chlamydia* lacks any annotated orthologs of quorum-sensing systems, which are typically composed of various integrated gene regulatory networks separate from the systems described here. Were *Chlamydia* to use such a system that required sufficient RBs to trigger it, then it would be at risk should its host cell be lysed. Therefore, *Chlamydia*, with its limited gene repertoire, may have once again evolved a simple means to effect a complicated outcome.

Exactly how these signal inputs are integrated requires further investigation. How do proteases selectively modify regulatory networks, and what are their targets? Does ClpC degrade, for example, AhpC? How are redox cues sensed and transmitted with precise timing? Is DacA activity, for example, modulated by redox? How does cyclic di-AMP interact with metabolism and stress responses? Does c-di-AMP, for example, bind and alter the activity of ClpC? How do changes in ATP levels influence regulatory decisions, including Clp unfoldase activity? How are sigma factor activities coordinated with these secondary signals? Addressing these questions will require shifting from descriptive studies to mechanistic and systems-level analyses, and the field is currently developing and deploying such systems to advance our understanding of these unique pathogens.
